# Analysis of the Boston Waters

**Published:** 1846-04

**Authors:** 


### ANALYSIS OF THE BOSTON WATERS.

Preparatory to supplying the city of Boston with pure water, steps have been taken by the commissioners in charge of the work to have the water of the various ponds and rivers in the neighborhood of the city subjected to a rigid chemical analysis. The report of Mr. B. Silliman, Jr., on these waters, a copy of which is before us, is a very able document, and must prove an invaluable guide to the commissioners. After giving the quantity of every foreign ingredient discovered in each of the waters, the author details the results of his experments undertaken with the view of ascertaining their action upon leaden pipes. He found that *pure* water acted on the lead more than any other, which is in accordance with what chemists had oserved before, that saline matters protect lead to a great extent against the action of water. He observed also, that the water most abundant in carbonic acid, and which contained more than an average amount of solid matter, "had no effect whatever on the lead." These facts, as he states, "certainly appear anomalous, and lead to the conclusion that we are yet without the means of establishing any general rule by which we may judge whether any given water will act on lead; and that actual experience in every case is required before the question can be decided." His practical inference is, that, considering the deadly nature of lead poison, and the fact that the metal is dissolved by so many natural waters, "it is the course of safety to avoid, as far as possible, the use of lead pipes for conveying water which is to be used for drinking."

Mr. Silliman is devoting himself especially to analytical chemistry, in which useful walk of science he is already becoming distinguished.

